# Myosins XI-K, XI-1, and XI-2 are required for development of pavement cells, trichomes, and stigmatic papillae in *Arabidopsis*

**DOI:** 10.1186/1471-2229-12-81

**Published:** 2012-06-06

**Authors:** Eve-Ly Ojangu, Krista Tanner, Pille Pata, Kristel Järve, Carola L Holweg, Erkki Truve, Heiti Paves

**Affiliations:** 1 Department of Gene Technology, Tallinn University of Technology, Akadeemia tee 15, 12618, Tallinn, Estonia; 2 Nachhaltigkeits-Projekte, Alte Str. 13, 79249, Merzhausen, Germany

## Abstract

**Background:**

The positioning and dynamics of vesicles and organelles, and thus the growth of plant cells, is mediated by the acto-myosin system. In *Arabidopsis* there are 13 class XI myosins which mediate vesicle and organelle transport in different cell types. So far the involvement of five class XI myosins in cell expansion during the shoot and root development has been shown, three of which, XI-1, XI-2, and XI-K, are essential for organelle transport.

**Results:**

Simultaneous depletion of *Arabidopsis* class XI myosins XI-K, XI-1, and XI-2 in double and triple mutant plants affected the growth of several types of epidermal cells. The size and shape of trichomes, leaf pavement cells and the elongation of the stigmatic papillae of double and triple mutant plants were affected to different extent. Reduced cell size led to significant size reduction of shoot organs in the case of triple mutant, affecting bolt formation, flowering time and fertility. Phenotype analysis revealed that the reduced fertility of triple mutant plants was caused by delayed or insufficient development of pistils.

**Conclusions:**

We conclude that the class XI myosins XI-K, XI-1 and XI-2 have partially redundant roles in the growth of shoot epidermis. Myosin XI-K plays more important role whereas myosins XI-1 and XI-2 have minor roles in the determination of size and shape of epidermal cells, because the absence of these two myosins is compensated by XI-K. Co-operation between myosins XI-K and XI-2 appears to play an important role in these processes.

## Background

The size, shape and growth of plant organs are regulated by genetic and environmental factors [1]. There are several excellent systems in *Arabidopsis* to study epidermal cell development, root hairs, pavement cells, and trichomes are well-studied model systems to investigate the mechanisms of cell growth and morphogenesis [2]. Studies have shown that cytoskeletal dynamics, vesicle transport, small GTPase signaling and endoreduplication all play a role in the development of the specialized shapes of different epidermal cell types. Some mechanisms that determine cell shape and polarity are common between these cell types, while some remain specific to each [3].

Myosins are molecular motors that carry cargo along actin filaments. The actomyosin system plays a crucial role in regulating cellular structures and dynamics [4]. Phylogenetic analysis has revealed that the 17 myosin genes present in the *Arabidopsis* genome fall into two classes: class VIII containing 4 genes and class XI containing 13 genes [5-8]. Class VIII myosins are implicated in new cell wall formation, intercellular transport through plasmodesmata and endocytosis [9-13]. Immunolocalization and co-localization experiments have indicated that class XI myosins are involved in the movement of vesicles and organelles [14-17]. Studies using T-DNA mutant lines, RNA interference or overexpression of dominant-negative myosin forms have confirmed that particular class XI myosins are required for movement of Golgi stacks, mitochondria and peroxisomes [7,18-22]. A novel role in regulation of the actin cytoskeleton and ER dynamics has been shown for class XI myosins [22,23]. In addition, phenotype analysis of T-DNA insertional mutants in each of the 13 class XI myosins has shown that only two class XI myosins are important for normal development of specific epidermal cells: XI-K and XI-2 are required for the tip growth of root hairs and XI-K also plays a role in diffuse growth of trichomes [19,24]. Since mutants in only two of the 13 class XI myosin genes have a distinct phenotype, it has been proposed that the functions of class XI myosins are partially overlapping [19,20]. This hypothesis has been largely proven by phenotype analysis of double, triple and quadruple mutants, which showed that five class XI myosins (XI-1, XI-2, XI-K, XI-B and XI-I) exhibit varying degrees of functional redundancy in *Arabidopsis*[20,22]. Simultaneous inactivation of these myosins in triple and quadruple mutants influenced overall plant growth and fertility, affecting shoot development even more than root development. Triple and quadruple mutant lines exhibited dwarf rosette growth, reduced plant height, late flowering phenotype, reduced fertility and also reduced growth of roots and root hairs. It is now thought that vegetative development of *Arabidopsis* relies on the four myosins (XI-K, XI-2, XI-1, XI-I) and that organelle transport driven by these myosin motors is required both for polarized growth as well as for diffuse growth of plant cells [22].

Myosins represent only one of many different types of actin binding proteins. Actin binding proteins are specialized to regulate dynamics and organization of the actin cytoskeleton. Mutants of these proteins have a wide range of phenotypes. A common characteristic of these mutants is irregular expansion and shape of trichomes, leaf pavement cells, and epidermal cells of the hypocotyl and root [25-28]. A group of mutants, named *distorted*, were initially identified based on a distorted or irregular trichome phenotype. These plants carry mutations in genes coding actin polymerization regulating proteins, like components of ARP2/3 [29,30] and SCAR/WAVE complexes [31-34]. Trichomes of these mutants are smaller, bloated and misshapen due to aberrant expansion of the stalk and branches [29,30,33,35-38]. Two other phenotypically similar mutants, identified as weak *distorted* mutants are myosin mutant *xi-k* and the WD40/BEACH domain protein mutant *spirrig*. The trichome phenotype of *spirrig* mutants is weaker compared to other *distorted* mutants and the phenotype of *xi-k* in turn is weaker than that of *spirrig* mutants. Partial phenotypic overlap with *distorted* mutants indicated that XI-K and SPIRRIG could be involved in similar growth processes of certain epidermal cells as are ARP2/3 and/or SCAR/WAVE complex proteins [24,39].

To reveal the detailed functions of myosins XI-K, XI-1 and XI-2 in growth and development of epidermal cells we analyzed double and triple T-DNA insertional mutants of these myosins. The results of this current work show that these three myosins contribute to the development of different epidermal cells - not only to the growth of root hairs and leaf pavement cells, but also to the coordinated expansion of trichomes and elongation of the stigmatic papillae. Simultaneous depletion of all three myosins resulted in dwarf growth, delay in bolting and flower development and reduced fertility. Our results indicated that the reduced fertility of triple mutant plants was caused by delayed or insufficient development of floral organs. This manifested in insufficiently developed pistils that were not fully receptive for pollination. Our results also indicate that myosin XI-K plays a more important role in the determination of epidermal cell size and shape than the other two myosins examined.

## Results

### Myosins XI-1, XI-2 and XI-K have overlapping roles in regulating shoot size

**Figure 1:**
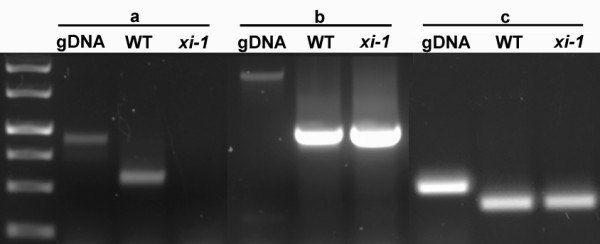
**RT-PCR analysis of the*XI-1*** **T-DNA insertional line Salk_022140.** Two different primer pairs were used to amplify two regions within the *XI-1* mRNA: **a** – primers spanning the T-DNA insertion site; **b** - primers for downstream region of the T-DNA insertion site; **c **- control amplified with *GAPB* primers. gDNA – genomic DNA; WT - wild type.

Double mutant lines *xi-1/xi-2*, xi-*1/xi-kxi-2/xi-k* and triple mutant line *xi-1/xi-2/xi-k* were generated, and the genotype combinations were identified by PCR. Single mutant lines used for crossings were analyzed by RT-PCR to confirm the presence or lack of myosin mRNAs in each mutant. When primers downstream of the T-DNA insertion site were used for RT-PCR, the respective myosin transcript was absent in *xi-2* (data not shown; see [19]), present in *xi-1* (Figure 1) and present in *xi-k* (data not shown; see [19,24]) mutants. When primers spanning the T-DNA insertion site were used, none of the mutant lines produced any transcript (see Figure 1 for *xi-1*). It has been shown previously that no XI-K protein is produced in the *xi-k* mutant, although the corresponding mRNA was present [19]. In *xi-1* mutant, the T-DNA is inserted into the coding region; thus, we assumed that no functional XI-1 protein can be produced in this single mutant as well as in respective double and triple mutant plants. A schematic diagram shows T-DNA insertions sites for mutant lines *xi-1xi-2* and *xi-k* (see Additional file 1).

**Figure 2:**
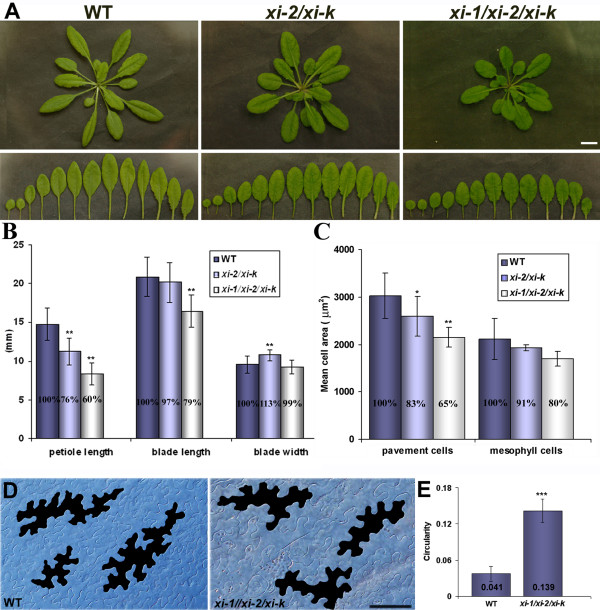
**Leaf and cell size. A**) Size of the rosette leaves of five-week-old plants. Rosette leaves of *xi-2/xi-k* and *xi-1/xi-2/xi-k* in comparison with wild type (WT). Leaves on the lower panel originate from the rosettes on upper panel. Bar = 10 mm. **B**) Mean size of leaf blades and petioles (±SD). The 5th to 10th rosette leaves were used for the size measurements. Note shorter petioles and smaller leaf blades of the *xi-2/xi-k* and *xi-1/xi-2/xi-k* plants in comparison with WT. Asterisks indicate statistical significance (** p < 0.01, Repeated Measures ANOVA with Dunn’s Multiple Comparisons Test, n = 18). **C**) Mean cell areas of pavement and mesophyll cells of five-week-old rosette leaves (±SD). In the case of *xi-2/xi-k* and *xi-1/xi-2/xi-k* the cell area is smaller compared to WT (* p < 0.05 and ** p < 0.01, One-Way ANOVA with Dunnett’s Multiple Comparisons Test). **D**) Shape of pavement cells of leaf abaxial epidermis. Note less extended lobes of the *xi-1/xi-2/xi-k* pavement cells in comparison with WT (black cells on the image). Bar = 50 μm. **E**) Circularity values of pavement cells on leaf abaxial epidermis (mean ± SD). Circularity reflects the ratio of pavement cell area to the perimeter. Cells with values near one are more circular, meaning that *xi-1/xi-2/xi-k* cells have more round morphology than WT cells (*** p < 0.001, unpaired *t*-test with Welch correction, n = 13-16).

To uncover the possible functions of myosins XI-1, XI-2 and XI-K in development of various epidermal cell types, we analyzed the overall phenotype of respective double and triple mutant plants. *xi-2/xi-k* double mutants had a slightly decreased rosette size while *xi-1/xi-2/xi-k* triple mutants showed more reduced rosette size (Figure 2A). Rosette size of all single mutants and the double mutants *xi-1/xi-2* and *xi-1/xi-k* was similar to that of wild type and was not investigated further. Rosettes of *xi-2/xi-k* and *xi-1/xi-2/xi-k* were investigated in more detail and both the leaf size as well as the cell area of rosette leaves was measured. Leaf size measurements showed that average length of *xi-2/xi-k* leaf blade was similar to wild type and for *xi-1/xi-2/xi-k* it was 21% (p < 0.01) smaller (Figure 2B; Additional file 2). The average width of the leaf blade of *xi-2/xi-k* and *xi-1/xi-2/xi-k* was comparable with wild type. The average length of *xi-2/xi-k* and *xi-1/xi-2/xi-k* petioles was 24% (p < 0.01) and 40% (p < 0.01) shorter than in wild type, respectively (Figure 2B; Additional file 2). Cell size measurements revealed that pavement cell area of *xi-2/xi-k* and *xi-1/xi-2/xi-k* plants was reduced by 17% (p < 0.05) and 35% (p < 0.01) and mesophyll cell area by 9% and 20%, respectively (Figure 2C, Additional file 3). In addition, we found that pavement cells of *xi-1/xi-2/xi-k* leaves had slightly less expanded lobes than those of wild type (Figure 2D). Circularity as a quantitative descriptor of cell shape complexity has been used for characterization of several cell types in plants. A perfect circle has the circularity equal to 1.0 and a cell with many deep lobes would have the circularity closer to 0 [40-42]. To characterize the shape of pavement cells, Image-J based circularity value of wild type and *xi-1/xi-2/xi-k* cells of the 5th and 6th rosette leaf was calculated. The average circularity of wild type and *xi-1/xi-2/xi-k* pavement cells was 0.041 and 0.139 (p < 0.0001), respectively (Figure 2E, Additional file 4), meaning that the pavement cells of the triple mutant are more round.

The results confirmed that myosins XI-1, XI-2 and XI-K have overlapping roles in shoot development, and that myosins XI-K and XI-2 are more important in this process than the XI-1.

We also noted that both bolt formation and onset of flowering of *xi-1/xi-2/xi-k* plants was delayed for two weeks on average (Additional file 5). In addition, the flowering time of *xi-2/xi-k* plants was occasionally delayed for about a week (data not shown). The average height of the inflorescence shoots of *xi-2/xi-k* and *xi-1/xi-2/xi-k* plants was reduced 9% and 17% (p < 0.001), respectively, when compared to wild type (Additional file 6). In addition, the growth of root hairs of double and triple mutant plants was decreased in a similar manner as described previously [20,22] (data not shown).

### Distorted trichome phenotype of *xi-k* is amplified in double and triple mutant plants

**Figure 3:**
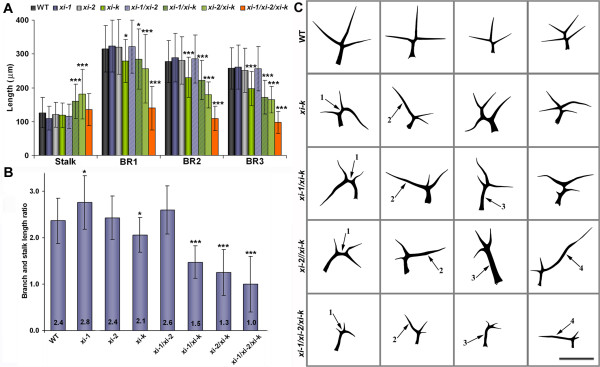
**Size and morphology of leaf trichomes.A**) Stalk and branch length of leaf trichomes (mean ± SD): BR1 – first branch; BR2 – second branch; BR3 – third branch; WT – wild type. **B**) Ratios of mean lengths of trichome branches and stalk (±SD). In WT, *xi-1*, *xi-2*, *xi-k* and *xi-1/xi-2* the stalk length constitutes approximately half of the branch length. In *xi-1/xi-k* and *xi-2/xi-k* the stalk is abnormally increased and in *xi-1/xi-2/xi-k* the branches are equal or slightly longer than stalk. Asterisks indicate statistical significance (* p < 0.05 and *** p < 0.001, Kruskal-Wallis with Dunn’s Multiple Comparisons Test, n = 73-109). **C**) Morphology of leaf trichomes. Numbers and arrows show: (1) trichomes with elongated interbranch zone, (2) unproportionally elongated individual branches, (3) unproportionally elongated stalks, (4) sword-shaped trichomes. Note also that modest bending of trichomes is visible in the case of all mutants. The number of trichomes with irregular shape was quantified by counting the presence of at least one of the phenotypes. Bar = 300 μm.

We have previously shown that myosin mutant *xi-k* partially phenocopies the mild trichome phenotype of *distorted* mutants [24]. Therefore leaf trichomes of myosin double and triple mutant plants were examined. Two parameters were followed to characterize trichomes - cell size and shape. For the size analysis, the length of trichome branches and the height of stalk was measured. The branch length and stalk height in *xi-1* and *xi-2* was similar to wild type (Figure 3A, Additional file 7). The average length of *xi-k* trichome branches was 83% of wild type but the height of the stalk was similar to wild type (Figure 3A, Additional file 7). Size measurements showed that trichomes of *xi-1/xi-2* were comparable with wild type. Trichome branch length of *xi-1/xi-k* was 79% of wild type and comparable with *xi-k*. The length of trichome branches of *xi-2/xi-k* and *xi-1/xi-2/xi-k* plants was 70% (p < 0.001) and 40% (p < 0.001) of wild type, respectively (Figure 3A, Additional file 7). In contrast, trichome stalks of *xi-1/xi-k* and *xi-2/xi-k* were abnormally elongated compared to wild type. The average height of trichome stalks in *xi-1/xi-k*, and *xi-2/xi-k* were 126% (p < 0.001) and 143% (p < 0.001) of wild type, respectively (Figure 3A, Additional file 7). The stalk height of *xi-1/xi-2/xi-k* trichomes was similar to wild type. The calculation of the branch and stalk length ratios revealed that in wild type, *xi-1xi-2xi-k* and *xi-1/xi-2* the stalk height constituted approximately half of the branch length (ratio values from 2.1 to 2.8; Figure 3B; Additional file 8). In *xi-1/xi-k* and *xi-2/xi-k*, the trichome stalks were abnormally elongated, and the height of trichome stalk constituted 70% (p < 0.001) and 90% (p < 0.001) of branch length, respectively (length ratios between 1.3 and 1.5; Figure 3B; Additional file 8). In the case of triple mutant, the length of trichome branches was decreased dramatically and the average height of trichome stalk was equal or slightly longer than the branch length (ratio value below 1, p < 0.001; Figure 3B; Additional file 8).

The number of trichomes with irregular shape was quantified by measuring the frequency at which several types of differences from wild type trichome shape occured. The differences we measured were: differences in branch positioning (elongated interbranch zone), individual branch length, stalk height or bended shape of the trichome (Figure 3C). The number of irregular trichomes on leaves of *xi-1*, *xi-2* and *xi-1/xi-2* plants was similar to wild type (values from 5% to 8%). In *xi-k* plants, 22% of leaf trichomes exhibited an irregular phenotype. Trichomes on the leaves of the double mutants *xi-1/xi-k*, *xi-2/xi-k* and the triple mutant *xi-1/xi-2/xi-k* were more irregular than those on the single mutant *xi-k*. Specifically, in the double mutant *xi-1/xi-k*; approximately 38% of leaf trichomes showed irregular phenotype compared to *xi-k*. Very characteristic for *xi-1/xi-k* was the appearance of trichomes with abnormally elongated stalks, a phenotype not found in the single *xi-k* mutant (Figure 3C). Trichome phenotype of *xi-k* was more severe in *xi-2/xi-k* and *xi-1/xi-2/xi-k* plants, where 56% and 90% of trichomes were irregular and frequently exhibited both abnormally elongated stalks as well as abnormally elongated single branches (sword-shaped trichomes) (Figure 3C, Additional files 7 and 8). In the case of *xi-1/xi-k, xi-2/xi-k* and *xi-1/xi-2/xi-k* plants, the bent shape of trichomes was more frequent than in *xi-k*.

**Figure 4:**
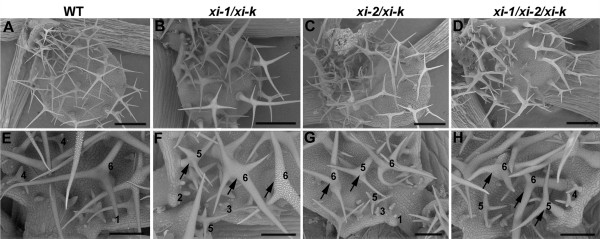
**Morphology of leaf trichomes at different developmental stages. A-D**) Overall view of trichomes of the two-week-old leaves. Bar = 250 μm. **E-H)** Close-up view of trichomes. Numbers indicate the different developmental stages of trichomes. Arrows indicate the irregular shape or elongation of stage 5 and 6 trichome branches of *xi-1/xi-k*, *xi-2/xi-k* and *xi-1/xi-2/xi-k* plants. Bar = 100 μm.

We used scanning electron microscopy to monitor stalk and branch expansion defects during trichome development. Trichome development is divided into six stages based on morphological features. Stage 4 and 5 trichomes were investigated because branches are formed in stage 4 and rapid branch elongation begins in stage 5 [35,43]. We did not detect significant differences between stage 4 trichomes of double mutant, triple mutant and wild type plants. Differences between wild type and mutant trichomes, irregular elongation and modest bending of stalk and branches, were clear in late stage 5 or stage 6 trichomes of *xi-1/xi-kxi-2/xi-k*, and *xi-1/xi-2/xi-k* plants (Figure 4A-H).

The phenotype of double mutants *xi-1/xi-k, xi-2/xi-k* and triple mutant *xi-1/xi-2/xi-k* all show more severe mutant phenotypes than any single mutant, suggesting that all three myosins participate in elongation of trichome stalks and branches. Among these mutants *xi-1/xi-k* had the weakest and *xi-1/xi-2/xi-k* had the strongest phenotype. These results indicate that myosin XI-K contributes more significantly than XI-1 and XI-2 to the trichome development because the absence of both myosins can be compensated by XI-K. Myosin XI-2 in turn plays a more important role in the trichome expansion than XI-1, as the phenotype of *xi-2/xi-k* was stronger than that of *xi-1/xi-k*.

### Simultaneous depletion of myosins XI-1, XI-2 and XI-K influences the shape of trichome nuclei

Wild type trichomes undergo approximately four rounds of endoreduplication during maturation leading to a three to four branched cell with an average DNA content of 32 C (32 times the DNA content of the haploid genome) [44-47]. It has been found that mutants with smaller trichomes contain less DNA, whereas mutants with increased cell size were found to have additional endoreduplication rounds [46]. To test whether the smaller size of myosin mutant trichomes could be related to the ploidy level, we quantified the nuclear DNA of fully mature trichomes on 14 days old plants using Hoechst staining. Confocal scanning fluorescence microscopy measurements showed that the ploidy level of the single, double and triple mutant trichomes was similar to wild type, average DNA content being 32 C (data not shown), suggesting that trichomes of these mutants undergo four successive endoreplication cycles during maturation, as in wild type.

**Figure 5:**
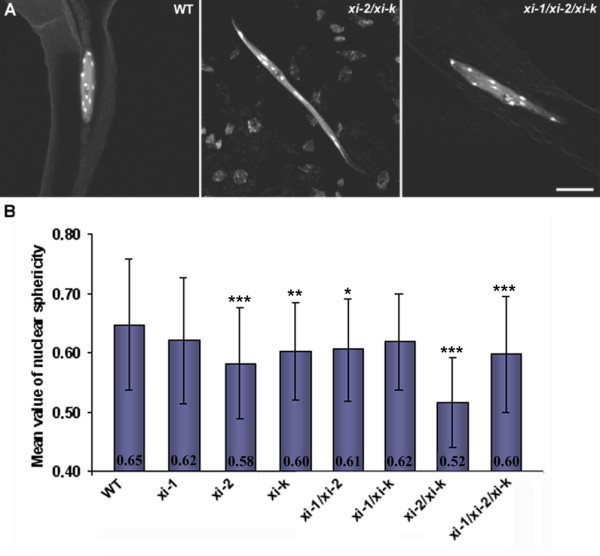
**Shape of leaf trichome nuclei. A**) Hoechst stained nuclei of two-week-old leaf trichomes. Abnormally elongated nuclei of *xi-2/xi-k* and *xi-1/xi-2/xi-k* trichomes are shown. Bar = 15 μm. **B**) Sphericity of trichome nuclei (mean ± SD). Sphericity is the ratio of the volume of a nucleus to the surface area of the nucleus, which is the smallest in *xi-2/xi-k* mutants. Asterisks indicate statistical significance (* p < 0.05, ** p < 0.01, and *** p < 0.001, Kruskal-Wallis with Dunn’s Multiple Comparisons Test, n = 55-157).

Although we did not detect differences in ploidy level, we found that the nuclei of mutant trichomes exhibited a different shape compared to wild type. Trichomes of all single mutants and double mutants *xi-1/xi-2* and *xi-1/xi-k* had spherically shaped nuclei, similar to wild type. Trichomes of *xi-2/xi-k* and *xi-1/xi-2/xi-k* frequently had abnormally elongated nuclei (Figure 5A). Quantification analysis revealed that the average sphericity of wild type trichome nuclei was 0.65 and that of *xi-1*, *xi-2*, *xi-k, xi-1/xi-2* and *xi-1/xi-k* nuclei was between 0.58 and 0.62 (Figure Inter/InternalRef>, Additional file 9). The average sphericity of *xi-2/xi-k* and *xi-1/xi-2/xi-k* trichomes was 0.52 (p < 0.001) and 0.60 (p < 0.001), respectively (Figure 5B, Additional file 9). Taken together, nuclei of wild type trichomes had the most circular shape and nuclei of double mutant *xi-2/xi-k* had the most elongated shape, indicating that co-operation between myosin XI-K and XI-2 is necessary for normal nuclear morphology in trichomes. Since both the irregular trichome shape and the sphericity of nucleus were most dramatically expressed in *xi-2/xi-k* background, a correlation analysis between these phenotypic features in *xi-2/xi-k* was performed. The calculated Pearson’s correlation coefficient r = −0.7120 (p < 0.0001; n = 32) shows that smaller nuclear sphericity values (i.e. nucleus is elongated) are correlated with mutant trichome phenotype (Additional file 10).

### Simultaneous depletion of myosins XI-1, XI-2 and XI-K influences the growth of floral organs and fertility

**Figure 6:**
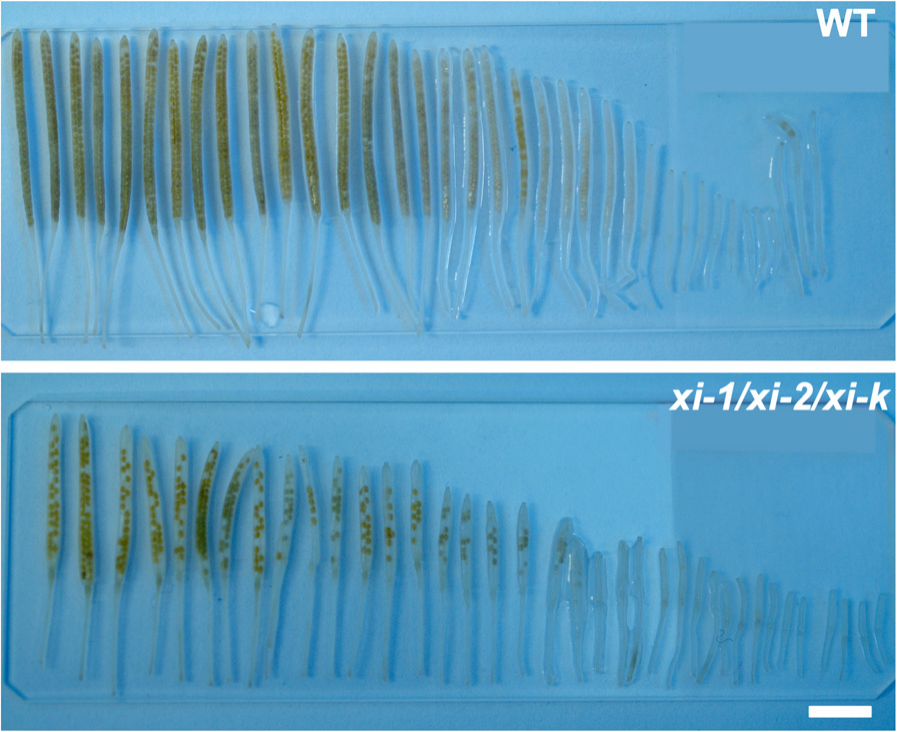
**Length of siliques of the primary shoot.** In *xi-1/xi-2/xi-k*, siliques are significantly shorter, containing much less seeds than those in wild type (WT). Bar = 5 mm.

To assess the fertility of the analyzed double and triple mutant lines, the number and length of the siliques per plant was measured and the number of seeds per silique was counted. Fertility of single and double mutant lines was comparable to wild type and was therefore not investigated further. For *xi-1/xi-2/xi-k*, variations in the number of normally and abnormally developed siliques were significant (siliques with length from 4 to 10 mm were assessed as abnormal). As an average, *xi-1/xi-2/xi-k* plants had up to 60% (p < 0.001) of abnormally developed siliques on main stem, when compared to wild type (Figure 6, Additional file 11).

**Figure 7:**
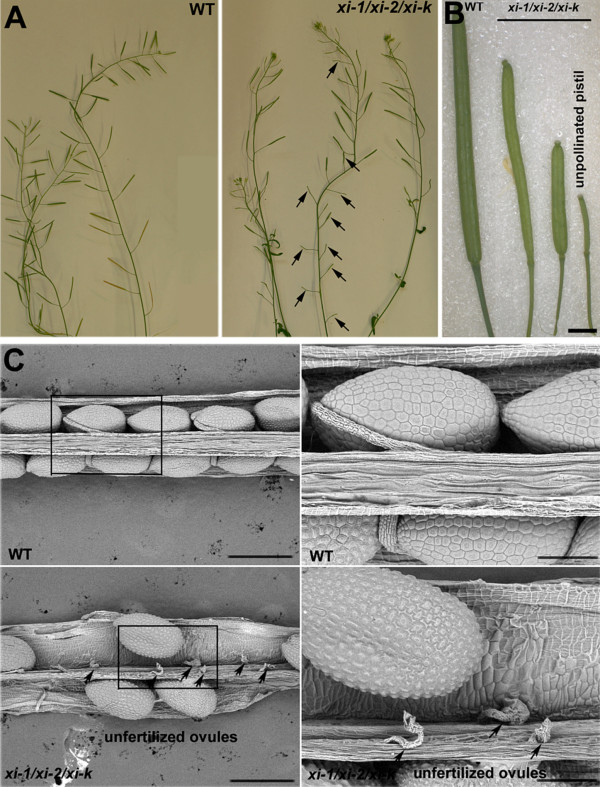
**Silique size and seed development. A**) Shoots of the eight-week-old plants. Arrows indicate the unpollinated pistils on the primary stem of *xi-1/xi-2/xi-k*. **B**) Representative wild type (WT) and *xi-1/xi-2/xi-k* siliques are shown. Note the heterogenous development of triple mutant siliques on the main stem. Bar = 2 mm. **C**) Scanning electron micrographs of developing seeds in manually opened siliques. Arrows point to the unfertilized ovules in *xi-1/xi-2/xi-k* siliques. Left panel, bar = 500 μm; right panel, bar = 150 μm.

Moreover, after onset of flowering, up to 60% (p < 0.001) of flowers emerged on the primary shoot of *xi-1/xi-2/xi-k* plants remained mostly seedless because the pistils remained unpollinated or were poorly pollinated (Figure 7A-B, Additional file 11). The average length of these siliques was 6.5 mm (p < 0.001) containing an average of only two (p < 0.001) fertilized ovules (Figure 7A-B; Additional file 12). This process continued approximately two to three weeks after bolting. About three weeks after bolting, there was a “switch” and plants started to produce siliques only slightly underdeveloped or normal in size with average length 12.9 mm (Figure 7A-B, Additional file 12). These siliques contained up to 28% (p < 0.0001) of unfertilized ovules (Figure 7C, Additional file 12). The number of unpollinated pistils, underdeveloped and normal siliques varied to a great extent between the *xi-1/xi-2/xi-k* plants. These variations in silique size were prevalent on primary shoots and less on axillary shoots of *xi-1/xi-2/xi-k* plants. In wild type, occasionally the first two flowers on the inflorescence remained seedless and the number of underdeveloped siliques was up to 5% (Additional file 12).

Next, anthers and pistils were examined to identify whether the decreased fertility of triple mutant was caused by defects in male or female reproductive organs. For this, Alexander’s staining of pollen, in vitro growth assays and aniline blue staining of pollen tubes was performed. Alexander’s staining showed that the viability of *xi-1/xi-2/xi-k* pollen grains was similar to wild type (Additional file 13). Using in vitro pollen tube growth assay we could not detect differences between triple mutant and wild type pollen tube growth (data not shown). These results indicated that the decreased fertility of *xi-1/xi-2/xi-k* triple mutant plants was not dependent on pollen viability or on the ability of the pollen to form a pollen tube.

**Figure 8:**
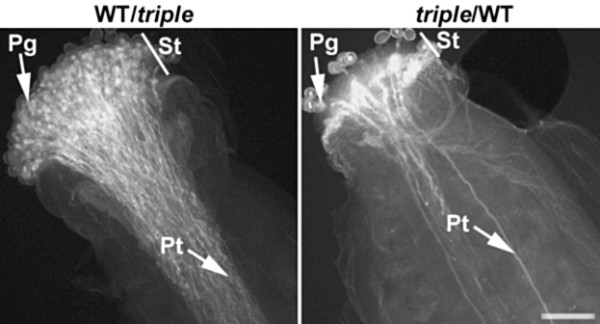
**Development of pollen tubes.** Pollen tube growth was followed in cross-pollinated pistils: WT/*triple* – wild type (WT) pistil was pollinated with *xi-1/xi-2/xi-k*; *triple*/WT – *xi-1/xi-2/xi-k* pistil was pollinated with WT. Note the number of pollen grains attached to the stigmas and the number of pollen tubes formed. In the case of *triple*/WT, WT pollen grains were not able to attach effectively on the surface of *xi-1/xi-2/xi-k* stigma and only few pollen tubes were formed. Aniline blue staining of pollen tubes was performed 12 hours after hand-pollination. Pg – pollen grains, St – stigma, Pt – pollen tubes. Bar = 50 μm.

Cross-pollination of wild type and *xi-1/xi-2/xi-k* pistils was performed. Aniline blue staining of pollinated pistils demonstrated that when wild type pistils were pollinated with *xi-1/xi-2/xi-k* pollens (WT/*triple*), the growth of pollen tubes in pistils was similar as in self-pollinated wild type (Figure 8). WT/*triple* siliques contained only 2-5% unfertilized ovules counted 9 days after pollination. Inversely, when *xi-1/xi-2/xi-k* pistils were pollinated with wild type pollen (*triple*/WT), pistils developed very heterogeneously. In some cases, the fertilization was normal, but often wild type pollen grains could not attach effectively to the surfaces of *xi-1/xi-2/xi-k* stigmas and form pollen tubes (Figure 8). As a result, *triple*/WT pistils often remained poorly pollinated, no siliques or shorter siliques were formed and a variable amount of ovules (12-53%) in shorter siliques remained unfertilized. We observed a similar phenotype both in cross-pollinated *triple*/WT as well as in self-pollinated *xi-1/xi-2/xi-k* pistils (Additional file 14). Reciprocal crosses between the wild type and triple mutant *xi-1/xi-2/xi-k* revealed that the reduced fertility of triple mutant is female reproductive tract specific.

**Figure 9:**
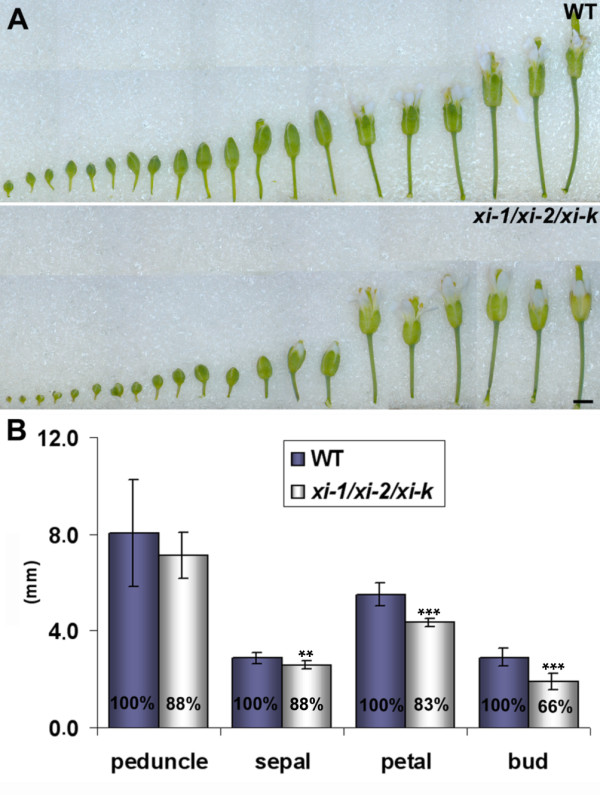
**Size of floral organs. A**) Developmental stages of flowers from inflorescence of the primary shoot are shown. Note the smaller size of *xi-1/xi-2/xi-k* flowers and flower buds. Bar = 1 mm. **B**) Mean length of peduncles, sepals, petals and flower buds (±SD). Opened flowers and flower buds of the inflorescences (from main stems) were selected for measurements. The average size of floral organs of *xi-1/xi-2/xi-k* is smaller than in wild type (WT). Asterisks indicate statistical significance (* p < 0.05, ** p < 0.01, and *** p < 0.001, unpaired *t*-test with Welch correction, n = 6-12).

Making reciprocal crosses it seemed that floral organs of *xi-1/xi-2/xi-k* are significantly smaller than those of wild type and the size and architecture of flowers was therefore examined in more detail (Figure 9A). Indeed, measurements of floral organs showed that *xi-1/xi-2/xi-k* flowers were smaller compared to the wild type (Figure 9B, Additional file 15). The length of peduncles, sepals and petals of *xi-1/xi-2/xi-k* flowers was 88%, 88% (p < 0.01) and 83% (p < 0.001) of wild type, respectively. The length of *xi-1/xi-2/xi-k* flower buds was only 66% (p < 0.001) of wild type (Figure 9B, Additional file 15).

**Figure 10:**
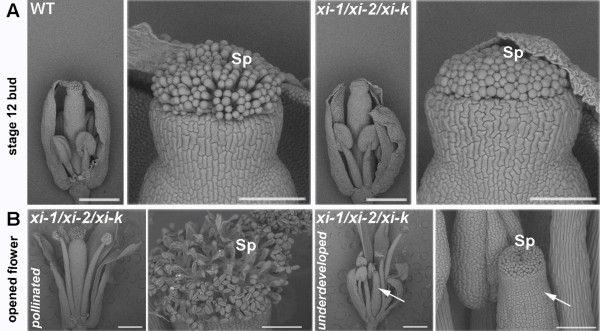
**Flower development of*xi-1/xi-2/xi-k*. A**) Scanning electron micrographs of stage 12 flower buds of wild type (WT) and *xi-1/xi-2/xi-k*. Epidermal surface of the stigma has papillar structure. Note that stigmatic papillae (Sp) of *xi-1/xi-2/xi-k* are shorter than those of WT. **B**) Scanning electron micrographs of stage 14 flowers of *xi-1/xi-2/xi-k*. As *xi-1/xi-2/xi-k* flowers develop heterogeneously, both well developed (pollinated) as well as underdeveloped flower is shown*.* In the case of underdeveloped flower the development of whole pistil is retarded. Arrows indicate the retarded growth of the pistil, arrested in developmental stage 10. On the left panel: overall view of the flower. Bar = 500 μm. On the right panel: close-up view of the pistil. Bar = 100 μm. Sp – stigmatic papillae.

Next, the architecture of the triple mutant and wild type pistils was studied using scanning electron microscopy. Twenty stages of *Arabidopsis* flower development have been distinguished [41]. Floral buds just before pollination (stage 12) and opened flowers (stage 13 or 14) were examined because wild type pistils of these developmental stages are mature and receptive to pollination. We observed that the elongation of stigmatic papillae of stage 12 *xi-1/xi-2/xi-k* buds was delayed, remaining on the level of stage 10 or 11 stigmas (Figure 10A). In stage 13 and 14 flowers the development of *xi-1/xi-2/xi-k* stigmatic papillae varied from normal to stunted. We noticed that in some cases also *xi-1/xi-2/xi-k* pistil itself did not develop to the wild type level even during later stages of development (Figure 10B).

We suggest that the insufficient development of stigmatic papillae, which renders the stigma not fully receptive (mature) for pollination, is the main reason for the reduced fertility of *xi-1/xi-2/xi-k* triple mutant plants. These results indicate that myosins XI-1, XI-2 and XI-K together are needed for normal development of floral organs.

## Discussion

Characterization of double and triple mutant plants revealed that myosins XI-K, XI-1, and XI-2 have redundant functions not only in development of root hairs and shoots [19,20,22] but also in expansion of trichomes, lobe extension of pavement cells, and in elongation of stigmatic papillae. Our results showed that simultaneous depletion of these three myosins affects several types of epidermal cells thereby influencing the growth and size of leaves, inflorescences, floral organs and the fertility of the plant. Differences between most phenotypic features (dwarf growth, decreased cell size, delayed flowering time and reduced fertility) between triple mutant *xi-1/xi-2/xi-k* and wild type, described in this work are similar and comparable with results published by Peremyslov and coworkers [22], and here we add several new characteristics (disorders in the development of pavement cell lobes, trichome stalk and branches and stigmatic papillae). Results concerning rosette size, plant height and fertility of the double mutants *xi-1/xi-k* and *xi-2/xi-k* are inconsistent with previously published data [20]: we show here that the rosette size and shoot height of the *xi-1/xi-k* plants are similar to the wild type, and on the contrary, the rosette size and shoot height of *xi-2/xi-k* plants are decreased compared to the wild type. Thus, the mutant phenotype (rosette size, shoot height, onset of flowering, and size and shape of trichomes) of *xi-2/xi-k* plants is always stronger than that of *xi-1/xi-k* plants and the phenotype of *xi-1/xi-2/xi-k* triple mutant plants is always stronger than that of *xi-2/xi-k*. Double and triple mutant lines used in this work were different than those described previously [20,22]: different T-DNA insertional lines of the *xi-1* (Salk_022140) and *xi-2* (Sail_632_D12) but the same for *xi-k* (Salk_067972) were used by us to generate double and triple mutant lines. It is possible that the inconsistency of the shoot phenotype of double mutants can be explained by use of different T-DNA insertional lines for generating double and triple mutant lines. Other aspects like differences in laboratory conditions can cause these differences as well.

The size of plant leaves is determined by a combination of cell number, cell size and intercellular space (reviewed in [48]). Experiments with mericlinal *Nicotiana* chimeras and *Arabidopsis* brassinosteroid receptor mutants have shown that the leaf epidermis has a crucial role in regulating leaf size through influencing mesophyll cell number and cell size [48-50]. We show that double mutant *xi-2/xi-k* and triple mutant *xi-1/xi-2/xi-k* have smaller and less lobed epidermal cells than wild type, and in addition, these leaves are smaller than wild type. Thus, although the primary effect of the depletion of myosins XI-K, XI-1, and XI-2 in the leaf blade may be on epidermal cell size and shape, this ultimately causes smaller leaves, especially in the triple mutant.

Proper organisation of the actin cytoskeleton is important in coordinating directed expansion of trichome branches and, as has been shown for several *distorted* group mutants [26,27,35,51]. The myosin *XI-K* mutant phenocopies mild trichome phenotype similar to the *distorted* group mutants [24]. However, the irregular trichome phenotype has not been found in the rest of the single mutants of class XI myosin genes. Our characterization of the double and triple mutant plants revealed, however, that myosins XI-1, XI-2 and XI-K have redundant functions in the elongation of trichome stalks and branches. Our results indicated that myosin XI-K has a leading role and XI-1 and XI-2 have minor roles in trichome development, whereas myosin XI-2 contributes to the trichome expansion more than XI-1.

We found also that the irregular size of myosin double and triple mutant trichomes was independent of endoreduplication events. This is also the case in *distorted* mutants [52], which define genes coding actin polymerization regulating proteins, like components of ARP2/3 [29,30] and SCAR/WAVE complexes [31-34]. Although the ploidy level of mutant trichomes did not change, we found that nuclear morphology was markedly affected in double mutant *xi-2/xi-k* trichomes. The abnormally elongated shape of *xi-2/xi-k* nuclei correlated with the mutant trichome phenotype. It is known that nuclear dynamics and morphology occurs in a cell specific manner and is influenced by cell shape and nuclear DNA content [53]. In plant cells the nucleus is positioned within a basket of dense actin filaments connected to the transvacuolar strands and cortical cytoskeleton [54-56]. Recently it has been shown that simultaneous depletion of myosins XI-1, XI-2 and XI-K caused defects in organization of actin filaments in root hairs and in the cells of leaf midvein epidermis [22]. Moreover, Ueda et al. [23] demonstrated that myosin XI-K in co-operation with myosin XI-2 is involved in organizing actin bundles and ER network in epidermal cells of cotyledonary petioles. In addition, transient expression in onion cells showed that GFP-fused head-neck domain of XI-2 had an increased fluorescence signal specifically near the nucleus [57]. Taking this into consideration, it is very likely that the organization of actin filaments is affected in the trichomes of *xi-1/xi-kxi-2/xi-k* and *xi-1/xi-2/xi-k* plants. It remains to be resolved how myosins XI-K and XI-2 regulate the organization of the nucleus-associated actin bundles, and thus nuclear shape, during trichome development.

Mutations in genes coding various actin related proteins in plants lead to defects in actin filament organization. For example, the organization of cortical actin filaments in the majority of *distorted* mutants is affected [32-34,52,58]. Comparing the phenotypes of myosin double and triple mutant plants and those of group *distorted* mutants revealed apparent similarity in the trichome phenotype of the *xi-1/xi-2/xi-k* with *pirogi* and *xi-2/xi-k* with *spirrig* plants [31,39]. It should be noted that the overall phenotype severity of *xi-2/xi-k* and *xi-1/xi-2/xi-k* plants was weaker than those of *pirogi* and *spirrig* mutants. For example, gaps between adjacent cells in cotyledons and hypocotyls, typical for *distorted* mutants, were not apparent in the analysed myosin mutants. On the other hand, typical characteristics for *xi-1/xi-kxi-2/xi-*k and *xi-1/xi-2/xi-k* trichomes like abnormally elongated trichome stalks have not been reported in *distorted* mutants. The phenotypic similarity between myosin mutants and *distorted* mutants also suggests, that the organization of actin filaments could be affected in trichomes of *xi-1/xi-kxi-2/xi-k* and *xi-1/xi-2/xi-k*.

Changes in branch morphology during stage 4 of trichome development are obvious indicators of aberrant actin function in *distorted* mutants [32-34]. In myosin double and triple mutant plants the irregular trichome phenotype became apparent in late stage 5 or stage 6 trichomes. This reveals that class XI myosins are required for the trichome development during later stages of morphogenesis: during rapid growth of trichome branches or during trichome maturation, when the cell wall thickens and becomes covered with papillae.

Pavement cells of the triple mutant *xi-1/xi-2/xi-k* had less extended lobes. It is known, that the shape of pavement cells and the extension of lobes, depends also on the microfilament dynamics and that most of the *distorted* mutants display less lobed morphology of pavement cells [3,34,59]. Relying on the phenotypic overlap it is tempting to speculate that the functions of myosins XI-K, XI-2 and perhaps of XI-1 may be related to the mechanisms controlled by ARP2/3 or SCAR/WAVE proteins/complexes.

Reproduction in *Arabidopsis* is dependent on interactions between pollen grains and papillar cells on the surface of the stigma [60]. The stigma is an epidermal structure composed of papillae, i.e. bulbous elongated cells. In the mature pistil the papillae are properly extended and form elongated cells receptive to the recognition, attachment and germination of pollen grains [61,62]. It has been shown that fewer pollen grains can adhere to immature stigmas and germinate. Immature stigmas are able to promote pollen tube growth to some extent, but the immature pistils are often unable to guide pollen tubes to the ovules [60,62,63]. Our current results indicated that the triple mutant *xi-1/xi-2/xi-k* has major deviations in the effectiveness of fertilization. We showed that the reduced fertility of *xi-1/xi-2/xi-k* was caused by delayed or insufficient development of the stigmatic papillae, making pistils less receptive for pollination. This indicates that class XI myosins are required for proper development of *Arabidopsis* pistils and therefore for fertilization.

Apical-basal patterning of *Arabidopsis* gynoecium is auxin dependent, and crosstalk between the actin cytoskeleton and auxin signaling is well known [64-66]. There are little data available concerning the actin cytoskeleton during pistil development. It has been shown that actin filaments of stigmatic papillae of self-incompatible *Brassica rapa* are differentially organized before and after pollination and that these changes in actin dynamics are associated with pollen hydration and germination [67]. We suggest, that the class XI myosins in the stigmatic papillae may fulfill a similar role as in other tip growing cell types [19,20,24].

In *Arabidopsis* not only stigmatic papillae, but also other epidermal cell types like part of the transmitting tract and the integument of the ovules have the same developmental origin, the meristematic epidermal L1 layer [61]. We do not exclude that defects in these cells may also influence the fertility of the *xi-1/xi-2/xi-k*, as the flowers of triple mutant were smaller than those of wild type and occasionally exhibited retarded growth with completely underdeveloped pistils. Our results indicate that all three myosins (XI-K, XI-2, XI-1) together are required for normal development of *Arabidopsis* shoots and floral organs.

## Conclusion

We conclude that (1) myosins XI-1, XI-2 and XI-K have partially redundant roles in the growth of different epidermal cells (pavement cells, trichomes, stigmatic papillae), (2) myosin XI-K has more important role and myosins XI-1 and XI-2 have minor roles in these growth processes, (3) cooperation between myosins XI-K and XI-2 appears to be important for maintaining normal growth of epidermal cells and thus the size of plant organs. We conclude that the decreased size and delayed or insufficient development of floral organs affects the fertility of myosin mutants.

## Methods

### Plant material and growth conditions

*Arabidopsis thaliana* (ecotype Columbia-0) seeds of *xi-1* (Salk_022140; At1g17580), *xi-2* (Sail_632_D12; At5g43900) and *xi-k* (Salk_067972; At5g20490) T-DNA mutant lines were obtained from the Nottingham Arabidopsis Stock Centre [68]. Homozygous single mutant lines of *xi-k* (Salk_067972; previously known by allele name *XIk-2*) and *xi-2* (Sail_632_D12) have been described earlier [19,24]. Homozygous single mutant lines were used to generate the double mutant lines *xi-1/xi-2xi-1/xi-kxi-2/xi-k* and triple mutant line *xi-1/xi-2/xi-k.* Homozygous plants of the double and triple mutant were identified by two PCRs. In the first PCR, a pair of gene-specific primers designed to anneal on either side of the T-DNA insertion were used, which in case of homozygosity does not produce a band of the predicted size. In the subsequent PCR, the T-DNA border specific primer and primers of the first PCR were used. To confirm the location of the T-DNA insertion in the respective myosin gene, the PCR products were sequenced. Double and triple mutant lines used in this work were partially different than those described previously [20,22]: different T-DNA insertional lines of the *xi-1* and *xi-2* were used to generate double and triple mutant plants.

Seeds from single, double and triple mutant lines were harvested from plants of the same age and stored at least three weeks in the dark at 4°C. Cold stratified seeds were soaked in water at 4°C for 1–4 days and sowed directly in soil (Biolan OY) containing 50% (w/v) of vermiculite. Plants were grown in Sanyo growth chambers at 22 ± 2°C and 60% of relative humidity under long day (16-h light) photoperiod.

### RNA isolation and RT-PCR

RNA was extracted from two-week-old seedlings and DNase I (Ambion) treated as described by Oñate*-*Sánchez and Vicente-Carbajosa [69]. For first-strand synthesis, RevertAid Premium Reverse Transcriptase (Fermentas) and Random Hexamer Primer mixture (Fermentas) were used according to manufacturer’s instructions. Equivalent amounts of cDNA template were used for amplification of fragments of *XI-1XI-2* and *XI-K* mRNAs. Two pairs of gene specific primers were used for each single mutant: one pair spanning the T-DNA insertion site and the other pair downstream of the insertion site. In all cases constitutively expressed B subunit of chloroplast glyceraldehydes-3-phosphate dehydrogenase (*GAPB*, At1g42970) specific primers were used to quantify mRNA levels. Primers used for RT-PCR of the *XI-1* and *GAPB* are listed here:

XI-1_022140_LP (5′-AGTCCAGAAGAATTTCCGCCG-3′),

XI-1_022140_RP (5′-GCCTGTCAATTTCGTTGCTCA-3′),

XI-1_3000_ Fw (5′-GCAATTGAAGAAGCAAGTTCAGTTAAT-3′),

XI-1_3900_ Rev (5′-CAGAGGGGAAATCTCTTTCTTCATCTT-3′),

GAPB_Fw (5′-CTTAACATATAGTTGTTCATCAGAC-3′),

GAPB_Rev (5′-GCGCCTCTTGTCTCTGTTGAC-3′).

Sequencing reactions were performed with BigDye Terminator Cycle Sequencing Kit (Applied Biosystems) according to the protocol provided by the manufacturer and analyzed with DNA analyzer ABIPRISM^TM^ 3130 (Applied Biosystems).

### Cell area and circularity measurements

Five-week-old rosette leaves (5th and 6th leaf) were fixed in Carnoy’s fixative (ethanol:acetic acid, 3:1), washed with 70% ethanol and mounted in Hoyer’s mounting medium (30 g gum arabic, 50 ml distilled water, 200 g chloral hydrate, 16. ml glycerol).

Images of pavement and mesophyll cells were captured using differential interference contrast (DIC) microscopy by Olympus BX61 microscope with a 20x or 40x objective. For calculating cell areas the total image area was divided with total cell number per image using Adobe Photoshop 7.0.

To quantify the differences in cell shape, circularity of pavement cells (from abaxial side) was measured using ImageJ software (http://rsb.info.nih.gov/ij/). Cell circularity was calculated according to the formula 4π*area/perimeter^2^[41]. For measuring circularity, black and white binary images of single pavement cells were used. Binary images were generated using Adobe Photoshop 7.0.

### Trichome isolation and analysis

Intact trichomes from mature leaves were isolated as described by Zhang and Oppenheimer [70]. Toluidine blue stained trichomes were mounted in Mowiol medium (6 g glycerol; 2.4 g Mowiol 4–88; 6 ml distilled water; 12 ml 0.2 M Tris buffer pH 8.5). Images taken with a 4x or 10x objective of the Olympus BX61 were processed with Adobe Photoshop 7.0. For trichome size analysis, the height of stalks and branches was measured using Image Tool 3.0 (http://ddsdx.uthscsa.edu/dig/itdesc.html). The number of trichomes with irregular shape was quantified by counting the presence of at least one of the following phenotypes: elongated interbranch zone, unproportionally elongated individual branches, abnormally elongated stalks, sword-shaped trichomes or slightly twisted shape of trichomes.

For the ploidy and sphericity analysis of trichome nuclei, two-week-old soil grown seedlings were fixed in Carnoy’s fixative, washed three times with distilled water (3x15 minutes) and stained overnight with 1 μg/ml of Hoechst 33342 (Molecular Probes). Samples were washed three times with water (3x15 minutes) and mounted in Mowiol medium. Hoechst fluorescence was visualized with 100x objective and 405 nm excitation of laser scanning microscope (Carl Zeiss LSM 510 DUO). Images were quantified using Imaris (Bitplane Scientific Software). Sum of the fluorescence isosurfaces of Hoechst stained nuclei was used for calculation of ploidy levels of wild type and mutant trichomes; at least 50 trichome nuclei per experiment were measured. To calculate total DNA content the fluorescence of trichome nuclei was calibrated using guard cell nuclei, which are considered to be strictly diploid. In mature wild type trichome the total DNA content is 32 C, equal with the four rounds of endoreduplication.

Sphericity of trichome nuclei was calculated using fluorescence isosurfaces of Hoechst stained trichome nuclei. Sphericity (Ψ) of a nucleus is the ratio of the surface area of a sphere (with the same volume as the given nucleus) to the surface area of the nucleus [71].

To perform correlation analysis, sphericity of trichome nuclei was measured and the overall trichome phenotype was evaluated. Trichomes with normal phenotype (equal to wild type) and mutant phenotype were arbitrarily assigned values of 0 and 1, respectively. The phenotype was considered mutant if the trichome exhibited any of the following features: elongated interbranch zone, irregular branch length, abnormally elongated stalk and sword-shaped trichome.

### Analysis of fertility

For floral organ measurements inflorescences of mutant and wild type plants were dissected using double-sided tape, fine needle (G27) and tweezers. Images were captured using stereomicroscope (Zeiss SteREO Discovery V8) and measurements were done using ImageJ software. Fertility was evaluated measuring the length of siliques, counting the number of siliques and the number of developing seeds in siliques.

### Alexander’s staining of pollens

To stain mature pollen grains, flowers were collected from adult plants and stored in 10% ethanol for at least 2 hours at room temperature. Dehisced anthers were mounted into a drop of Alexander’s stain (10 ml of 95% ethanol, 10 mg Malachite green (1 ml of 1% solution in 95% alcohol), 50 ml of distilled water, 25 ml of glycerol, 5 g of phenol, 5 g of chloral hydrate 50 mg of acid fuchsin (5 ml of 1% solution in water), 5 mg of Orange G (0.5 ml of 1% solution in water) and 2 ml of acetic acid) [72]. A coverslip was placed on the anthers and the slides were incubated overnight at room temperature. Images were taken with a digital camera (Olympus DP70) installed on Olympus BX61 microscope with a 20x objective.

### Aniline blue staining of pollen tubes

For pollen tube staining, pistils were opened longitudinally 12 h after pollination and staining was performed as described by Pagnussat et al. [73]. The pistils were fixed overnight in Carnoy’s fixative, cleared in 10% chloral hydrate at 65 °C for 5 minutes, washed with water, and softened with 1 M NaOH at room temperature, washed with 0.1 M K_2_HPO_4_ buffer (pH 10) and stained with 0,1% decolorized aniline blue (in 0.1 M K_2_HPO_4_ buffer) for 3 hours. Finally pistils were washed briefly with 0.1 M K_2_HPO_4_ buffer, mounted on a microscope slide using a drop of 80% glycerol. Aniline blue fluorescence was visualized with 10x or 20x objective of laser scanning microscope (Carl Zeiss LSM 510 DUO).

### SEM analysis of siliques and pistils

Siliques, flowers and flower buds were dissected on double-sided tape using fine needle (27 G) and tweezers. Images were captured with Hitachi TM-1000 tabletop scanning electron microscope.

### Statistical analysis

Statistical analysis was performed with Microsoft Excel and GraphPad InStat software (GraphPad Software Inc., La Jolla, CA). All data are expressed as mean ± standard deviation (SD). Datasets were first tested for normality using the Kolmogorov-Smirnov test, and an appropriate statistical test was chosen (indicated in Figure legends and Additional files). For all tests, two-sided p-values were calculated. Pearson’s correlation coefficient was calculated to determine relationship between the shape of trichomes and sphericity of their nuclei (Additional file 10).

## Authors’ contributions

EO participated in most of the experimental analyses and wrote the draft of the manuscript. KT did part of the sequencing, RT-PCR, helped with SEM and DIC images and made cell area measurements. PP made statistical analysis of the data. KJ made *xi-1/xi-k* double mutant line and participated in root hair measurements. CH shared T-DNA line of *xi-2* and participated in creating double mutant *xi-2/xi-k* line. ET participated in design of the study and in analysis of the data and helped to draft the manuscript. HP made images and analysis of trichome nuclei; made images of root hairs; participated in design of the study and in analysis of the data and helped to draft the manuscript. All authors read and approved the final manuscript.

## Supplementary Material

Additional file 1 **A schematic diagram of *XI-1, XI-2, and XI-K*genes with the positions of the T-DNA insertions.** Black boxes represent exons, black lines introns, and gray boxes represent 5' and 3′ untranslated regions. Above the corresponding T-DNA insertion sites are shown. Click here for file

Additional file 2 **Data for Figure** 2**B: size of thefive-week-oldrosette leaves (from 5th to 10th, mm).** Click here for file

Additional file 3 **Data for Figure **2**C: cell areas (μm²) of pavement and mesophyll cells offive-week-oldrosette leaves.** Click here for file

Additional file 4 **Data for Figure **2**E: circularity of pavement cells on the leaf abaxial epidermis.** Click here for file

Additional file 5 **Shoot size of six-week-old plants.** Wild type plants are on the left and *xi-1/xi-2/xi-k* plants are on the right. Both bolt formation as well as onset of flowering of *xi-1/xi-2/xi-k* plants delays significantly (two weeks). Click here for file

Additional file 6 Data for supplementing Additional file 5: inflorescence shoot height (cm). Click here for file

Additional file 7 **Data for Figure **3A**: lenght of trichome stalk and branches (μm).** Click here for file

Additional file 8 **Data for Figure **3B**: length ratios of the trichome stalk and branches.** Click here for file

Additional file 9 **Data for Figure **5B**: sphericity of trichome nuclei.** Click here for file

Additional file 10 **Data for figure **5B**: spherisity data and correlation between the sphericity of the trichome nucleus and the trichome shape.** Click here for file

Additional file 11 **Data for Figure **7**: number of siliques per main stem.** Click here for file

Additional file 12 **Data for Figures **6** and **7**: length of the siliques (mm) and number of seeds per silique.** Click here for file

Additional file 13 **Pollen viability assessed by Alexander’s staining method.** Pollen viability (purple-colored cytoplasm of pollen grains) is similar both in wild type (WT) as well as in all double and triple mutant plants. Bar = 100 μm. Click here for file

Additional file 14 **Development of pollen tubes in self-pollinated pistils.** Shortly after the onset of flowering first three flowers on the primary shoot were analyzed. A-C) Aniline blue staining of wild type (WT) pistils. D-F) Aniline blue staining of *xi-1/xi-2/xi-k* pistils. In *xi-1/xi-2/xi-k*, pollen grains were not attached to the stigmas and pollen tubes were not formed. Pg – pollen grains; Pt – pollen tubes. Bar = 100 μm. Click here for file

Additional file 15 ***Data for Figure ***9**: size of floral organs (mm), length of the peduncles, sepals, petals and buds.** Click here for file
