# Supplementary figures and images for: Myosins XI-K, XI-1, and XI-2 are required for development of pavement cells, trichomes, and stigmatic papillae in Arabidopsis
Source: BMC Plant Biol. 2012 Jun 6;12:81. doi: 10.1186/1471-2229-12-81 (PMC3424107; doi:10.1186/1471-2229-12-81)

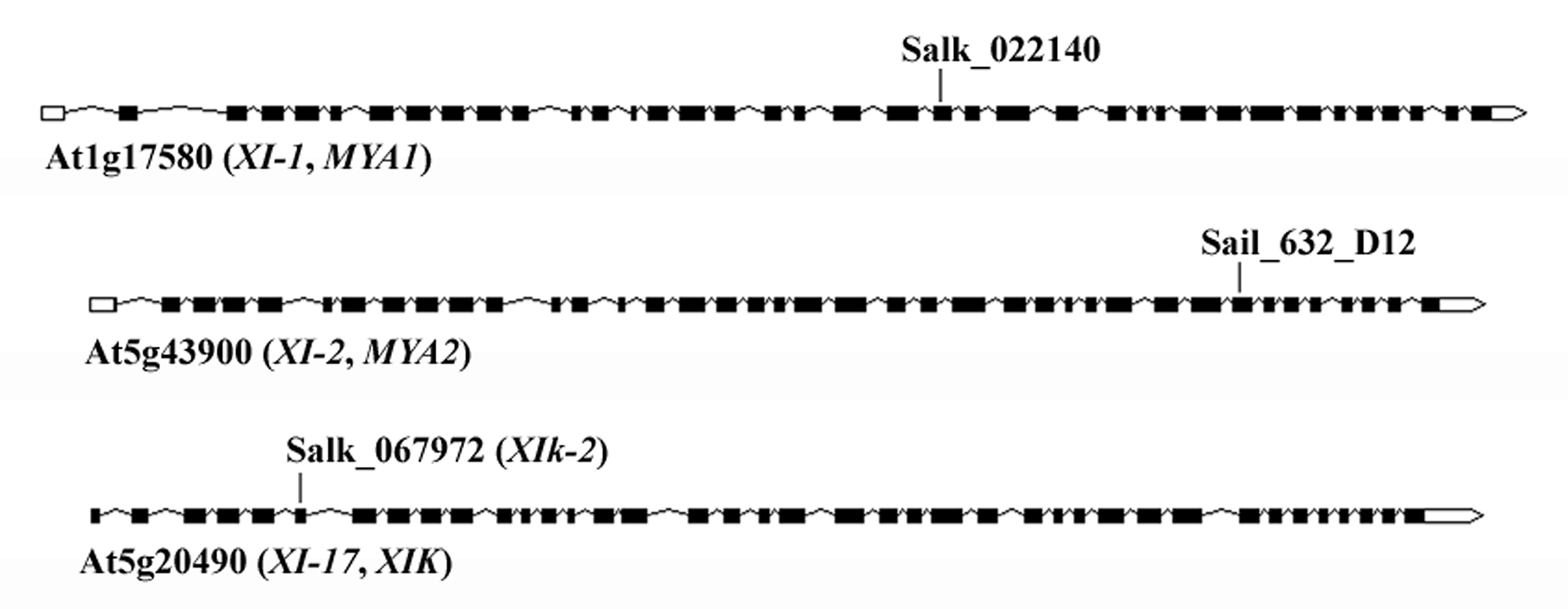

Supplement: Additional file 1 — A schematic diagram of XI-1, XI-2, and XI-Kgenes with the positions of the T-DNA insertions. Black boxes represent exons, black lines introns, and gray boxes represent 5' and 3′ untranslated regions. Above the corresponding T-DNA insertion sites are shown. [file 1471-2229-12-81-S1.jpeg]

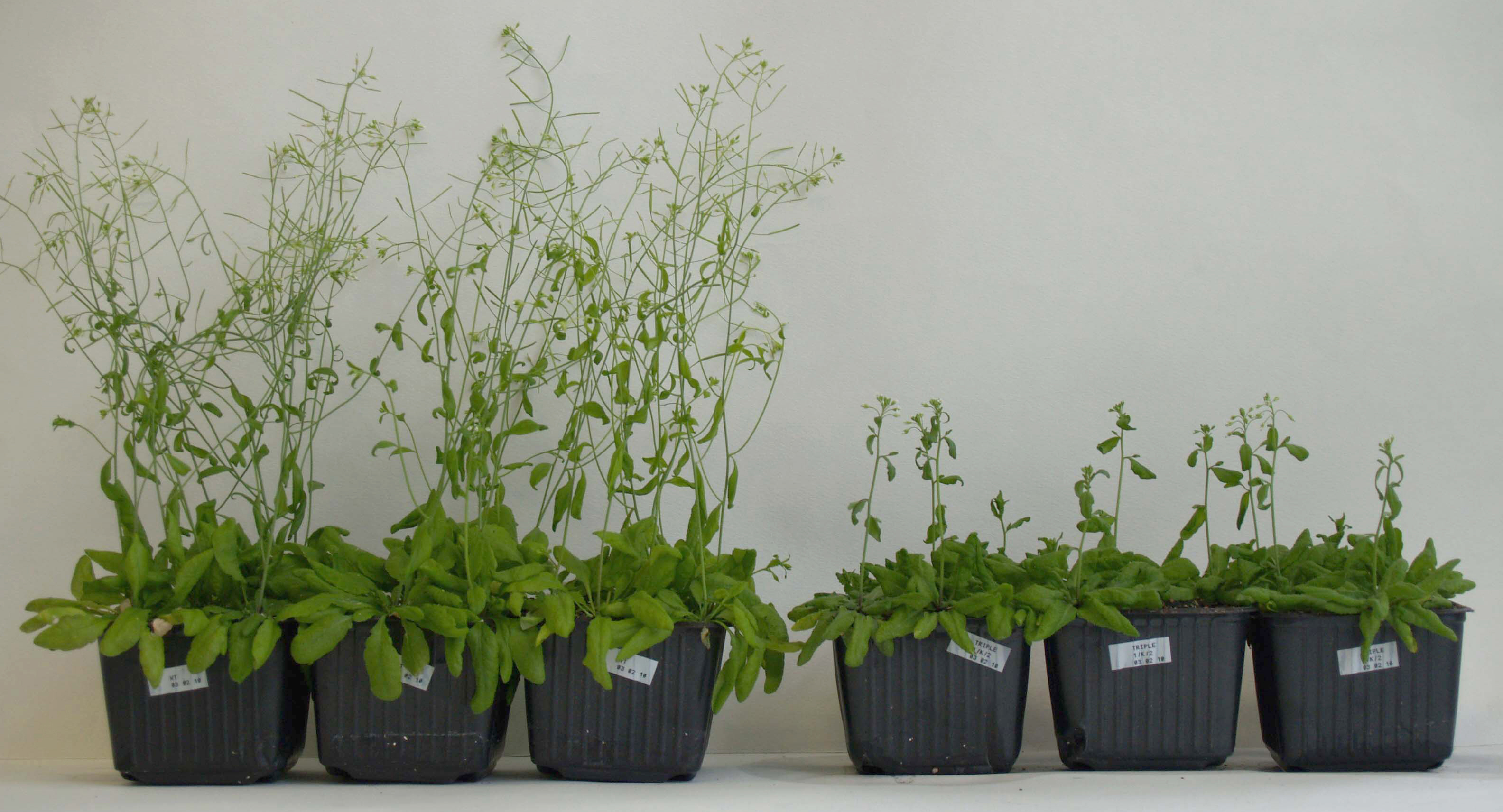

Supplement: Additional file 5 — Shoot size of six-week-old plants. Wild type plants are on the left and xi-1/xi-2/xi-k plants are on the right. Both bolt formation as well as onset of flowering of xi-1/xi-2/xi-k plants delays significantly (two weeks). [file 1471-2229-12-81-S5.jpeg]

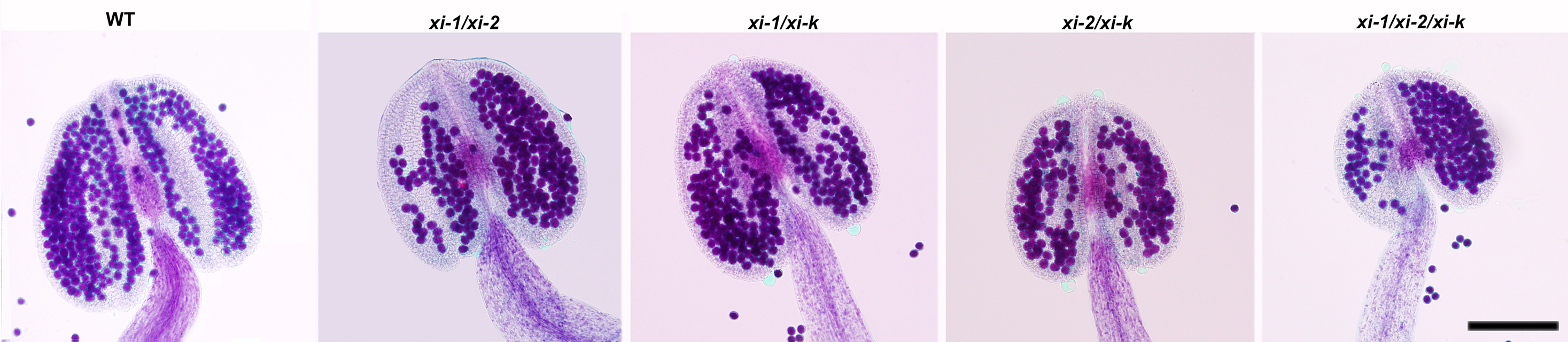

Supplement: Additional file 13 — Pollen viability assessed by Alexander’s staining method. Pollen viability (purple-colored cytoplasm of pollen grains) is similar both in wild type (WT) as well as in all double and triple mutant plants. Bar = 100 μm. [file 1471-2229-12-81-S13.jpeg]

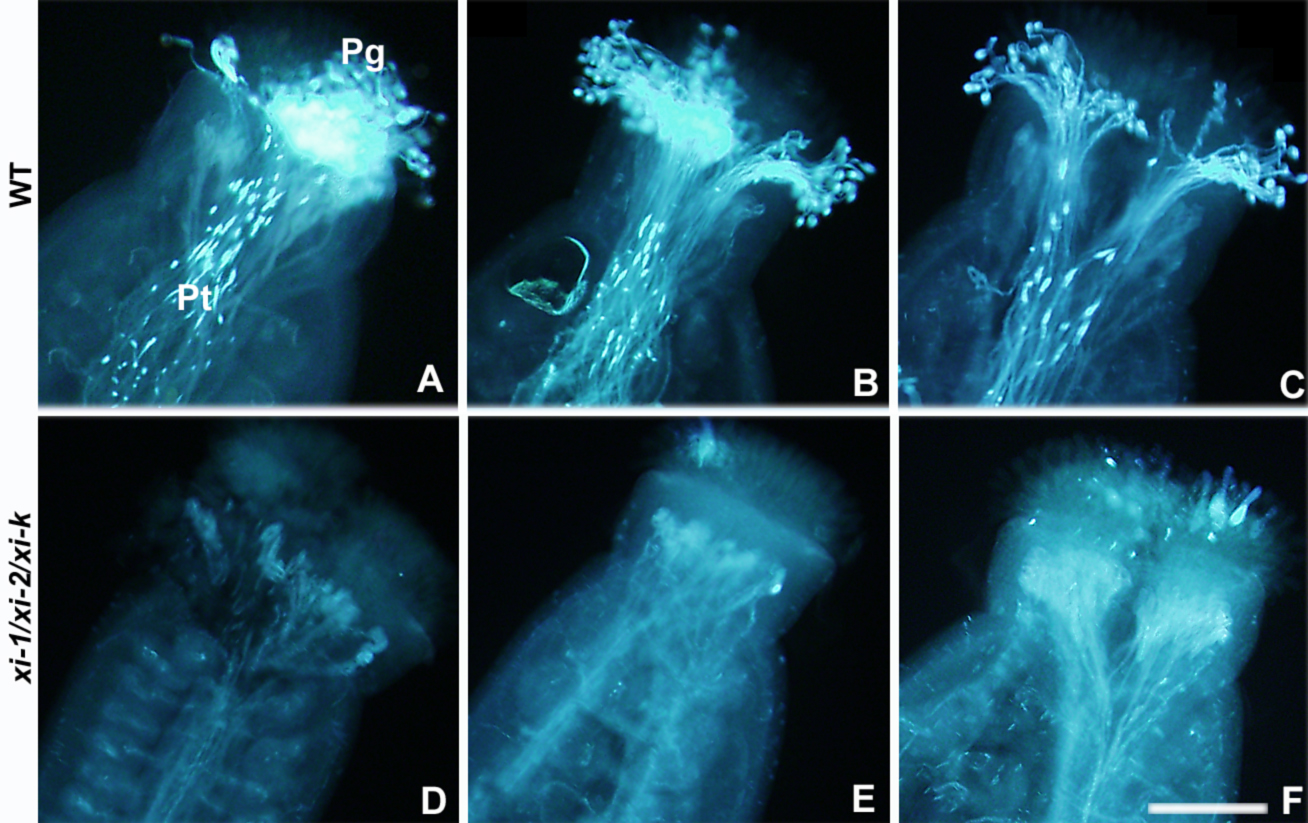

Supplement: Additional file 14 — Development of pollen tubes in self-pollinated pistils. Shortly after the onset of flowering first three flowers on the primary shoot were analyzed. A-C) Aniline blue staining of wild type (WT) pistils. D-F) Aniline blue staining of xi-1/xi-2/xi-k pistils. In xi-1/xi-2/xi-k, pollen grains were not attached to the stigmas and pollen tubes were not formed. Pg – pollen grains; Pt – pollen tubes. Bar = 100 μm. [file 1471-2229-12-81-S14.jpeg]
