# Supplementary material for: Myosins XI-K, XI-1, and XI-2 are required for development of pavement cells, trichomes, and stigmatic papillae in Arabidopsis
Source: BMC Plant Biol. 2012 Jun 6;12:81. doi: 10.1186/1471-2229-12-81 (PMC3424107; doi:10.1186/1471-2229-12-81)
Supplement: Additional file 2 — Data for Figure 2B: size of thefive-week-oldrosette leaves (from 5th to 10th, mm). [file 1471-2229-12-81-S2.pdf]

Additional file 2

Data for Figure 2B: size of the five-week-old rosette leaves (from 5th to 10th; mm).

	MEAN	MEDIAN	STDEV	SEM	n	Repeated Measures ANOVA	Dunn's test WT versus:	%
**petiole length**						P<0.0001		
**WT**	14.78	15.07	2.07	0.49	18			100
xi-2/xi-k	11.26	10.75	1.75	0.41	18		P<0.01	76
xi-1/xi-2//xi-k	8.84	9.10	1.26	0.30	18		P<0.01	60
**blade length**						P<0.0001		
**WT**	20.87	21.17	2.51	0.59	18			100
xi-2/xi-k	20.17	20.73	2.56	0.60	18		P>0.05	97
xi-1/xi-2//xi-k	16.58	17.30	2.16	0.51	18		P<0.01	79
**blade width**						P<0.0001		
**WT**	9.55	9.27	1.07	0.25	18			100
xi-2/xi-k	10.80	10.87	0.71	0.17	18		P<0.01	113
xi-1/xi-2//xi-k	9.48	9.57	0.82	0.19	18		P>0.05	99

Abbreviations: STDEV, standard deviation; SEM, standard error of the mean; n, number of data points.
Statistical analysis: Repeated Measures ANOVA and Dunn's Multiple Comparisons Test.
%: mean values of the wild type (WT) were arbitrarily set at 100% and compared to the mean values of the mutants.
