# Supplementary material for: Myosins XI-K, XI-1, and XI-2 are required for development of pavement cells, trichomes, and stigmatic papillae in Arabidopsis
Source: BMC Plant Biol. 2012 Jun 6;12:81. doi: 10.1186/1471-2229-12-81 (PMC3424107; doi:10.1186/1471-2229-12-81)
Supplement: Additional file 3 — Data for Figure 2C: cell areas (μm²) of pavement and mesophyll cells offive-week-oldrosette leaves. [file 1471-2229-12-81-S3.pdf]

Additional file 3

Data for Figure 2C: cell areas (μm²) of pavement and mesophyll cells of five-week-old rosette leaves.

***area**	**MEAN**	**MEDIAN**	**STDEV**	**SEM**	****n**	**One-Way Analysis ANOVA**	**Dunnett's test WT versus:**	**%**
**pavement cells**						P<0.0001		
**WT**	3382.7	3450.6	543.4	192.1	8			100
xi-2/xi-k	2820.4	2794.7	421.1	148.9	8		P<0.05	83
xi-1/xi-2/xi-k	2206.8	2214.6	204.8	72.4	8		P<0.01	65
**mesophyll cells**						P>0.05		
**WT**	2113.3	1954.9	434.9	217.5	4			100
xi-2/xi-k	1932.8	1931.2	63.2	31.6	4		P>0.05	91
xi-1/xi-2/xi-k	1699.8	1692.5	155.9	78.0	4		P>0.05	80

Abbreviations: STDEV, standard deviation; SEM, standard error of the mean; n, number of data points.
*Area measurement: the number of cells on the image divided by the image's area, whereas cells on the image over or touching the lines on top and on the left were counted, but cells over or touching the right or bottom lines were ignored.
**n indicates the number of images (each containing 50-80 cells) used for counting cells.
Statistical analysis: One-Way Analysis of Variance ANOVA and Dunnett's Multiple Comparisons Test.
%: mean values of the wild type (WT) were arbitrarily set at 100% and compared to the mean values of the mutants.
