# Supplementary material for: Myosins XI-K, XI-1, and XI-2 are required for development of pavement cells, trichomes, and stigmatic papillae in Arabidopsis
Source: BMC Plant Biol. 2012 Jun 6;12:81. doi: 10.1186/1471-2229-12-81 (PMC3424107; doi:10.1186/1471-2229-12-81)
Supplement: Additional file 4 — Data for Figure 2E: circularity of pavement cells on the leaf abaxial epidermis. [file 1471-2229-12-81-S4.pdf]

Additional file 4

Data for Figure 2E: circularity of pavement cells on the leaf abaxial epidermis.

	MEAN	MEDIAN	STDEV	SEM	n	unpaired *t*-test with Welch correction
***circularity**						P<0.0001
**WT**	0.041	0.037	0.013	0.003	16	
xi-1/xi-2/xi-k	0.139	0.142	0.019	0.005	13	

Abbreviations: STDEV, standard deviation; SEM, standard error of the mean; n, number of data points.

*Cell circularity was calculated according to the following formula $4\pi*\text{area}/\text{perimeter}^2$ using ImageJ software.

Statistical analysis: unpaired *t*-test with Welch correction.
