# Supplementary material for: Myosins XI-K, XI-1, and XI-2 are required for development of pavement cells, trichomes, and stigmatic papillae in Arabidopsis
Source: BMC Plant Biol. 2012 Jun 6;12:81. doi: 10.1186/1471-2229-12-81 (PMC3424107; doi:10.1186/1471-2229-12-81)
Supplement: Additional file 6 — Data for supplementing Additional file 5: inflorescence shoot height (cm). [file 1471-2229-12-81-S6.pdf]

Additional file 6

Data for supplementing Additional file 5: inflorescence shoot height (cm).

	**MEAN**	**MEDIAN**	**STDEV**	**SEM**	**n**	**Kruskal-Wallis test**	**Dunn's test WT versus:**	**%**
***shoot height**						P<0.01		
**WT**	31.00	32.00	5.05	1.03	24			100
xi-1/xi-k	29.35	28.00	4.28	0.84	26		P>0.05	95
xi-2/xi-k	28.21	27.50	4.36	0.82	28		P>0.05	91
xi-1/xi-2/xi-k	25.75	26.00	4.61	0.87	28		P<0.01	83

Abbreviations: STDEV, standard deviation; SEM, standard error of the mean; n, number of data points.

* Shoot heigth of eight week-old plants was measured (after flowering was complete for wild type).

Statistical analysis:Kruskal-Wallis Test and Dunn's Multiple Comparisons test.

%: mean values of the wild type (WT) were arbitrarily set at 100% and compared to the mean values of the mutants.
