# Supplementary material for: Myosins XI-K, XI-1, and XI-2 are required for development of pavement cells, trichomes, and stigmatic papillae in Arabidopsis
Source: BMC Plant Biol. 2012 Jun 6;12:81. doi: 10.1186/1471-2229-12-81 (PMC3424107; doi:10.1186/1471-2229-12-81)
Supplement: Additional file 7 — Data for Figure 3A: lenght of trichome stalk and branches (μm). [file 1471-2229-12-81-S7.pdf]

Additional file 7

Data for Figure 3A: lenghts of trichome stalk and branches (µm).

	MEAN	MEDIAN	STDEV	SEM	n	Kruskal Wallis test	Dunn's test WT versus:	%
**Stalk**						P<0.0001		
**WT**	126.5	117.9	44.1	4.5	97			100
xi-1	110.2	108.1	34.9	3.6	94		P>0.05	87
xi-2	120.6	114.5	36.9	3.5	109		P>0.05	95
xi-k	118.5	117.5	36.8	3.8	96		P>0.05	94
xi-1/xi-2	115.4	115.4	35.9	3.6	80		P>0.05	91
xi-1/xi-k	159.7	154.3	50.2	4.8	108		P<0.001	126
xi-2/xi-k	181.1	166.9	74.1	7.1	108		P<0.001	143
xi-1/xi-2/xi-k	136.0	132.4	47.7	5.5	73		P>0.05	107
**BR1**						P<0.0001		
**WT**	315.2	311.8	68.7	7.0	97			100
xi-1	323.7	324.7	76.7	7.9	94		P>0.05	103
xi-2	320.8	312.4	80.3	7.7	109		P>0.05	102
xi-k	279.7	274.2	63.8	6.5	96		P<0.05	89
xi-1/xi-2	322.2	322.2	78.5	7.8	80		P>0.05	102
xi-1/xi-k	284.7	272.8	88.5	8.5	108		P<0.05	90
xi-2/xi-k	256.7	232.9	101.1	9.7	108		P<0.001	81
xi-1/xi-2/xi-k	139.8	121.3	63.9	7.4	73		P<0.001	44
**BR2**						P<0.0001		
**WT**	278.0	268.9	61.9	6.3	97			100
xi-1	289.5	283.5	71.8	7.4	94		P>0.05	104
xi-2	280.9	277.4	70.6	6.8	109		P>0.05	101
xi-k	230.8	221.2	59.3	6.0	96		P<0.001	83
xi-1/xi-2	285.2	285.2	71.2	7.1	80		P>0.05	103
xi-1/xi-k	222.7	213.9	57.8	5.6	108		P<0.001	80
xi-2/xi-k	179.3	173.6	38.2	3.7	108		P<0.001	64
xi-1/xi-2/xi-k	108.9	104.8	35.7	4.1	73		P<0.001	39
**BR3**						P<0.0001		
**WT**	257.5	240.8	61.6	6.3	97			100
xi-1	261.0	260.2	65.2	6.7	94		P>0.05	101
xi-2	251.4	239.7	65.6	6.3	109		P>0.05	98
xi-k	197.3	191.4	50.1	5.1	96		P<0.001	77
xi-1/xi-2	256.2	256.2	65.4	6.5	80		P>0.05	99
xi-1/xi-k	171.9	162.5	50.2	4.8	108		P<0.001	67
xi-2/xi-k	165.2	159.5	39.4	3.8	108		P<0.001	64
xi-1/xi-2/xi-k	98.4	93.6	32.7	3.8	73		P<0.001	38

Abbreviations: BR1, branch 1; BR2, branch 2; BR3, branch 3; STDEV, standard deviation;
SEM, standard error of the mean; n, number of data points.

Statistical analysis: Kruskal-Wallis Test and Dunn's Multiple Comparisons Test.

%: mean values of the wild type (WT) were arbitrarily set at 100% and compared to the mean values of the mutants.
