# Supplementary material for: Myosins XI-K, XI-1, and XI-2 are required for development of pavement cells, trichomes, and stigmatic papillae in Arabidopsis
Source: BMC Plant Biol. 2012 Jun 6;12:81. doi: 10.1186/1471-2229-12-81 (PMC3424107; doi:10.1186/1471-2229-12-81)
Supplement: Additional file 8 — Data for Figure 3B: length ratios of the trichome stalk and branches. [file 1471-2229-12-81-S8.pdf]

Additional file 8

Data for Figure 3B: length ratios of the trichome stalk and branches.

	MEAN	MEDIAN	STDEV	SEM	n	Kruskal Wallis test	Dunn's test WT versus:
***BR/stalk**						P<0.0001	
**WT**	2.37	2.29	0.49	0.05	97		
xi-1	2.76	2.71	0.57	0.06	94		P<0.05
xi-2	2.43	2.39	0.47	0.04	109		P>0.05
xi-k	2.06	2.06	0.37	0.04	96		P<0.05
xi-1/xi-2	2.60	2.55	0.52	0.05	80		P>0.05
xi-1/xi-k	1.48	1.43	0.35	0.03	108		P<0.001
xi-2/xi-k	1.25	1.18	0.50	0.05	108		P<0.001
xi-1/xi-2/xi-k	1.00	0.86	0.60	0.07	73		P<0.001

Abbreviations: BR, trichome branch; STDEV, standard deviation; SEM, standard error of the mean; n, number of data points.

* BR/stalk ratio: average length of trichome branches (BR1, BR2, BR3) divided with the length of the stalk.

Statistical analysis: Kruskal-Wallis Test and Dunn's Multiple Comparisons Test.
