# Supplementary material for: Myosins XI-K, XI-1, and XI-2 are required for development of pavement cells, trichomes, and stigmatic papillae in Arabidopsis
Source: BMC Plant Biol. 2012 Jun 6;12:81. doi: 10.1186/1471-2229-12-81 (PMC3424107; doi:10.1186/1471-2229-12-81)
Supplement: Additional file 9 — Data for Figure 5B: sphericity of trichome nuclei. [file 1471-2229-12-81-S9.pdf]

Additional file 9

Data for Figure 5B: sphericity of trichome nuclei.

	MEAN	MEDIAN	STDEV	SEM	n	Kruskal-Wallis test	Dunn's test WT versus:
***Sphericity**						0.0001	
**WT**	0.65	0.67	0.11	0.009	157		
xi-1	0.62	0.64	0.11	0.013	69		P>0.05
xi-2	0.58	0.59	0.09	0.008	151		P<0.001
xi-k	0.60	0.60	0.08	0.010	75		P<0.01
xi-1/xi-2	0.61	0.61	0.09	0.012	55		P<0.05
xi-1/xi-k	0.62	0.62	0.08	0.009	81		P>0.05
xi-2/xi-k	0.52	0.51	0.08	0.008	88		P<0.001
xi-1/xi-2/xi-k	0.60	0.57	0.10	0.010	89		P<0.001

Abbreviations: BR, branch; STDEV, standard deviation; SEM, standard error of the mean; n, number of data points.

*Sphericity is a measure of how spherical (round) an object is, whereas values equal to 1.00 represent a perfect sphere.

Statistical analysis: Kruskal-Wallis Test and Dunn's Multiple Comparisons Test.
