# Supplementary material for: Myosins XI-K, XI-1, and XI-2 are required for development of pavement cells, trichomes, and stigmatic papillae in Arabidopsis
Source: BMC Plant Biol. 2012 Jun 6;12:81. doi: 10.1186/1471-2229-12-81 (PMC3424107; doi:10.1186/1471-2229-12-81)
Supplement: Additional file 10 — Data for figure 5B: spherisity data and correlation between the sphericity of the trichome nucleus and the trichome shape. [file 1471-2229-12-81-S10.pdf]

Additional file 10

Data for figure 5B: sphericity data and correlation between the sphericity of the trichome nucleus and the trichome shape.

**Sphericity**	**MEAN**	**MEDIAN**	**STDEV**	**SEM**	**n**	**Mann-Whitney test (sphericity):**	**r**	***p*-value (correlation):**
**WT**	0.83	0.84	0.07	0.01	56		N.A.*	
xi-2/xi-k	0.62	0.60	0.13	0.02	32	P<0.0001	-0.7120	P<0.0001

Abbreviations: BR, branch; STDEV, standard deviation; SEM, standard error of the mean; n, number of data points; r, Pearson's correlation coefficient; N.A., not applicable.

Statistical analysis: Mann-Whitney Test, Pearson's correlation coefficient.

* Can not be calculated as there is no variance in the shape of WT trichomes.
