# Supplementary material for: Myosins XI-K, XI-1, and XI-2 are required for development of pavement cells, trichomes, and stigmatic papillae in Arabidopsis
Source: BMC Plant Biol. 2012 Jun 6;12:81. doi: 10.1186/1471-2229-12-81 (PMC3424107; doi:10.1186/1471-2229-12-81)
Supplement: Additional file 11 — Data for Figure 7: number of siliques per main stem. [file 1471-2229-12-81-S11.pdf]

Additional file 11

Data for Figure 7: number of siliques per main stem of eight week-old plants.

	MEAN	MEDIAN	STDEV	SEM	n	Kruskal-Wallis test	Dunn's test WT versus:	%
**WT**								
**total n of siliques**	36.71	39.00	9.47	2.53	14	P>0.05		100
***n of abnormal siliques**	1.71	0.00	3.00	0.80		P<0.0001		5
xi-1/xi-k								
**total n of siliques**	32.75	30.50	7.85	1.96	16		P>0.05	100
**n of abnormal siliques**	0.13	0.00	0.50	0.13			P>0.05	0
xi-2/xi-k								
**total n of siliques**	33.77	31.00	7.04	1.95	13		P>0.05	100
**n of abnormal siliques**	0.00	0.00	0.00	0.00			P>0.05	0
xi-1/xi-2/xi-k								
**total n of siliques**	30.86	29.00	4.81	1.05	21		P>0.05	100
**n of abnormal siliques**	18.48	18.00	4.20	0.92			P<0.001	60

Abbreviations: STDEV, standard deviation; SEM, standard error of the mean; n, number of data points.
Statistical analysis: Kruskal-Wallis Test and Dunn's Multiple Comparisons Test.
*Siliques with the length from 4 to 10 mm were evaluated as abnormal.
%: mean number of siliques (per main stem) were taken as 100% and compared to the mean number of abnormal siliques.
