# Supplementary material for: Myosins XI-K, XI-1, and XI-2 are required for development of pavement cells, trichomes, and stigmatic papillae in Arabidopsis
Source: BMC Plant Biol. 2012 Jun 6;12:81. doi: 10.1186/1471-2229-12-81 (PMC3424107; doi:10.1186/1471-2229-12-81)
Supplement: Additional file 12 — Data for Figures 6 and 7: length of the siliques (mm) and number of seeds per silique. [file 1471-2229-12-81-S12.pdf]

Additional file 12

Data for Figures 6 and 7: length of the silique (mm) and number of seeds per silique.

	MEAN	MEDIAN	STDEV	SEM	n	Kruskal-Wallis test	Dunn's test WT versus:	Mann-Whitney test WT versus:
**WT**								
**silique length**	14.48	14.50	0.76	0.20	15	P<0.0001		
**seeds per silique**	57.60	56.00	2.80	0.72		P<0.0001		
**unfertilized ovules**	0.73	1.00	1.03	0.27		P<0.0001		
xi-1/xi-2/xi-k								
**silique length *I**	6.54	6.00	2.56	0.71	13		P<0.001	
**seeds per silique I**	2.15	1.00	2.91	0.81			P<0.001	
xi-1/xi-2/xi-k								
**silique length *II**	12.87	13.00	1.78	0.46	15		P>0.05	
**seeds per silique II**	50.93	51.00	4.22	1.09			P<0.05	
**unfertilized ovules II**	14.40	12.00	11.93	3.08				P<0.0001

Abbreviations: STDEV, standard deviation; SEM, standard error of the mean; n, number of data points.

Statistical analysis: Kruskal-Wallis Test, Dunn's Multiple Comparisons Test and Mann-Whitney Test.

*I - siliques developed after onset of flowering (during first two to three weeks) on main stem.

*II - siliques developed "after switch" (starting from three to four weeks after onset of flowering) on main stem.
