# Supplementary material for: Myosins XI-K, XI-1, and XI-2 are required for development of pavement cells, trichomes, and stigmatic papillae in Arabidopsis
Source: BMC Plant Biol. 2012 Jun 6;12:81. doi: 10.1186/1471-2229-12-81 (PMC3424107; doi:10.1186/1471-2229-12-81)
Supplement: Additional file 15 — Data for Figure 9: size of floral organs (mm), length of the peduncles, sepals, petals and buds. [file 1471-2229-12-81-S15.pdf]

Additional file 15

Data for Figure 9: size of floral organs (mm), length of the peduncles, sepals, petals and buds.

**Floral organs**		**MEAN**	**MEDIAN**	**STDEV**	**SEM**	**n**	**Unpaired *t*-test with Welch correction**	**%**
**WT**	**Peduncle**	8.05	8.77	2.21	0.84	7		100
	**Sepal**	2.94	2.88	0.24	0.08	10		100
	**Petal**	5.31	5.51	0.46	0.14	10		100
	**Bud**	2.91	2.91	0.36	0.10	12		100
xi-1/xi-2/xi-k	**Peduncle**	7.09	7.12	0.96	0.39	6	P>0.05	88
	**Sepal**	2.60	2.62	0.17	0.05	12	P<0.01	88
	**Petal**	4.40	4.36	0.44	0.13	12	P<0.001	83
	**Bud**	1.91	1.94	0.33	0.10	12	P<0.001	66

Abbreviations: STDEV, standard deviation; SEM, standard error of the mean; n, number of data points.

Statistical analysis: unpaired *t*-test with Welch correction.

%: mean values of the wild type (WT) were arbitrarily set at 100% and compared to the mean values of the mutants.
